# Computational identification and experimental validation of novel *Saccharum officinarum* microRNAs along with their targets through RT-PCR approach

**DOI:** 10.1080/15592324.2025.2452334

**Published:** 2025-01-28

**Authors:** Abdul Baqi, Samiullah, Jadoon Khan, Asma Sadiq, Yousaf Khan, Shahid Ali, Syed Nadeem ul Hassan Mohani, Naqeebullah Khan, Tawaf Ali Shah, Khalid S. Almaary, Youssouf Ali Younous, Mohammed Bourhia

**Affiliations:** a Department of Chemistry, University of Balochistan, Quetta, Pakistan; b Department of Allied Health Sciences, Sarhad University Islamabad Campus, Islamabad, Pakistan; c Department of Microbiology, University of Jhang, Faisalabad, Pakistan; d Department of Chemistry, COMSATS University Islamabad Campus, Islamabad, Pakistan; e Department of Microbiology, Quaid-I-Azam University Islamabad, Islamabad, Pakistan; f Department of Pharmacy, Sarhad University of Science and Technology, Islamabad, Pakistan; g College of Agriculture Engineering and Food Science, Shandong University of Technology, Jinan, China; h Department of Botany and Microbiology, College of Science, King Saud University, Riyadh, Saudi Arabia; i Evangelical College BP, N’Djamena, Chad; j Laboratory of Biotechnology and Natural Resources Valorization, Faculty of Sciences, Ibn Zohr University, Agadir, Morocco

**Keywords:** miRNA, *Saccharum officinarum*, RT-PCR amplification, psRNA target, WebLogo

## Abstract

Various metabolic and cell signaling processes impact the functions of sugarcane plant cells. MicroRNAs (miRNAs) play critical regulatory roles in enhancing yield and providing protection against various stressors. This study seeks to identify and partially characterize several novel miRNAs in sugarcane using *in silico* tools, while also offering a preliminary assessment of their functions. This was accomplished by predicting novel conserved miRNAs in sugarcane plants using a variety of genomics-based techniques like BLASTn, MFOLD, psRNA Target, sequence logo, Weblogo, primer-3, etc. and annotated using miRBase and NCBI. For validation, RT-PCR method was used along with agarose gel. After the preparation of fourteen randomly chosen primers, they were validated by RT-PCR. Accordingly, they contain fifty specific targeted proteins with substantial targets in the structural, transcriptional protein, etc. Furthermore, the sof-miR5025a directs the heat repeat protein while the voltage-dependent anion is governed by sof-miR8005a. Similarly, the sof-miR7768b and sof-miR6249b monitor the pathogenesis-related protein and zinc finger, C_2_H_2_ type protein, which assist as transcription factors. Thus, the novel sugarcane miRNAs target a wide range of important genes help regulate the environment for sugarcane to generate a higher-quality crop.

## Introduction

MicroRNAs (miRNAs) are small, endogenous RNA molecules ranging from 18 to 26 nucleotides (nt) in length. As a specific class of non-coding RNAs, miRNAs are thought to regulate gene expression either by promoting the cleavage of target mRNAs or by inhibiting their translation at the post-transcriptional level.^1^ MicroRNAs are generated from longer precursor molecules known as pre-miRNAs, which form stem-loop structures. These pre-miRNAs typically range from 70 to 500 nucleotides in length. In plants, Dicer-like 1 (DCL1) enzymes process these precursors by folding them into self-contained stem-loop secondary structures.^2,3^ DCL1 is responsible for processing microRNAs (miRNAs) from stem-loop precursor RNAs with partial double-stranded structures transcribed from MIR genes. DCL3, on the other hand, processes endogenous small interfering RNAs (siRNAs) originating from repeats and intergenic regions, with this activity relying on RNA-dependent RNA polymerase 2 (RDR2). DCL2 is involved in the antiviral response in plants infected with turnip crinkle virus, although it does not perform this role in plants infected with turnip mosaic virus or cucumber mosaic virus strain Y. Furthermore, it is indicated that DCL4 serves as the main processor for endogenous trans-acting siRNAs (tasiRNAs) that depend on RDR6. Molecular and phenotypic analyses of all *dcl* double mutants have further demonstrated partial compensatory roles among the DCL proteins. In cases where DCL4 is absent, some RDR6-dependent siRNAs are processed by DCL2 and DCL3, while in the absence of DCL3, certain RDR2-dependent siRNAs are generated by DCL2 and DCL4.^4^

Mature miRNAs monitor post-transcriptional levels of gene expression by directing mRNAs toward degradation or inhibiting protein translation. Indeed, the interaction of miRNAs with mRNAs depends on how well both complement each other.^5^ Expressed sequence tags (ESTs) strategies have been established to evaluate conserved miRNAs in plants,^6^ which play an important role in understanding the evolutionary relationship and conservancy of miRNAs among different species.^7^ EST-based approaches have several benefits over other computational and wet lab techniques^8–10^ because they can quickly identify novel conserved miRNAs during the non-availability of the whole genome in plants, timesaving, less laborious^10^ and could be confirmed through high-throughput sequencing tools.^11^

In plants, miRNAs almost always hybridize perfectly or almost perfectly with their targets, instructing the target mRNA degradation.^12^ According to a recent study, plant development processes like cell division, pressure response, absorption, irritation, and signal transduction all depend on miRNAs.^4,12,13^ Following that, an increasing number of studies involving miRNAs in animals, plants, and even viruses have been regularly found utilizing computational and experimental methodologies. According to the open-access database miRbase (Release 22), almost 48,860 miRNAs have been investigated from 271 species of plants and animals.^14^ miRNAs from varied plant species have been identified from various fully sequenced genomes, like 738 miRNAs from *Oryza sativa*, 525 from *Brachypodium distachyon*, 428 from *Arabidopsis thaliana*, 401 from *Populus trichocarpa*, 343 from *Solanum tuberosum*, 325 from *Zea mays*, 241 from *Sorghum bicolor*^14^ and 08 from *Thellungiella halophile*.^15^ The field of miRNA research is continuously growing, and a variety of computational and experimental strategies are being employed to profile and annotate miRNAs and their functions. These methods contain deep sequencing, direct cloning, and several other techniques. Comparative analysis of miRNA sequences across different plant species has revealed that some miRNAs are highly conserved from species to species, such as those present in mosses and eudicots of the plant kingdom.^13^ Comparative analysis of miRNA sequences across different plant species has been a powerful tool for studying miRNA biology for the identification of novel miRNAs in previously unexplored organisms. Comparative genome-based methodologies have recently been used to profile conserved miRNAs in many plant species. This includes *Z. mays*,^16,17^ *S*. *bicolor*,^18^ muskmelon^19^ and sugarcane.^20–27^

Sugarcane (*Saccharum officinarum*), a member of the grass family (Poaceae), is extensively planted, supplying almost 70% of the world’s sugar.^25^ Of all plants, sugarcane yields the most calories per growth unit. Sugarcane is used to make most of the sugar consumed worldwide. The sugarcane plant provides many other products besides sugar and the raw components required to make alcohol. Conventional sugar-producing techniques aim to enhance the sucrose amount and eliminate color by thermal and chemical methods of juice, syrup, and molasses.^26^ The present sugarcane cultivars are saccharum hybrids manufactured by crossing *S. spontaneum* with *S. officinarum* and then backcrossing with *S. officinarum*. In one study, *S*. *officinarum* comprised between 70% and 80% of the genetic makeup of hybrid saccharum species.^25^ The development of the plant family is dependent on genetically resistant cultivars, prolific seed production, modern agriculture, and the capacity to endure biotic and abiotic stress. It is possible to evaluate plant development by looking at its genetic makeup and seeding in different places.^27–29^

Over the past decade, research on sugarcane microRNAs (miRNAs) has advanced significantly, driven by their roles in essential biological processes, including growth regulation, stress response, and disease resistance. Enhanced capabilities in high-throughput sequencing and bioinformatics have allowed researchers to identify a growing number of miRNAs in sugarcane. The Sugarcane Genome Project has provided a valuable genomic foundation, aiding in the annotation and analysis of miRNAs in this complex polyploid species. Despite these advancements, sugarcane’s highly complex genome presents challenges for miRNA identification and characterization.

Specific sugarcane miRNAs have been found to affect key traits, such as biomass accumulation, tillering, and sucrose content, all of which are vital for optimizing yield and sugar production. Additionally, sugarcane miRNAs play crucial roles in the plant’s adaptation to environmental stresses, such as drought, salinity, and nutrient deficiency, which are critical for its resilience across diverse climates. Research has shown that miRNAs like miR393, miR396, and miR398 are involved in regulating stress-related pathways. In response to biotic stress, miRNAs contribute to the plant’s defense against pathogens, including fungi, bacteria, and viruses.

Through genetic engineering and biotechnology, researchers are working to enhance specific miRNAs in sugarcane to improve stress tolerance and promote growth. This gene-editing approach shows promising potential for developing sugarcane varieties with increased yield and resilience.^25,26^

Only 16 mature miRNAs are recorded in this bulky sugarcane food from the Poaceae family in the miRbase (http://www.mirbase.org/, Release 22: January 2019), a database of miRNAs. Our findings will also help characterize and comprehend novel sugarcane miRNAs more thoroughly. However, it is vital to profile more conserved miRNAs supporting these valuable grain crops. This research has profiled new sugarcane miRNAs and their targets using a precise comparative genome-based homolog search.

## Materials and methods

### Obtaining source miRNA sequences

With the assistance of miRbase, a library of miRNAs (http://www.mirbase.org/, Release 22: January 2019), the plant precursor and mature miRNA sequences were obtained.^14^ These reference miRNAs were acquired from 12 plant species like *Asparagus officinalis* (aof), *Aegilops tauschii* (ata), *Arabidopsis thaliana* (ath), *Brachypodium distachyon* (bdi), *Citrus sinensis* (csi), *Hordeum vulgare* (hvu), *Nicotiana tabacum* (nta), *Oryza sativa* (osa), *Sorghum bicolor* (sbi), *Solanum tuberosum* (stu), *Triticum aestivum* (tae), and *Zea mays* (zma). In order to anticipate fresh well-preserved miRNAs from the sugarcane-expressed sequences tags (ESTs), the obtained miRNAs sequences were utilized as the source miRNAs.

### Retrieving of potential miRNAs

Assuming the distinctive conserved sugarcane miRNAs through a comparative homology-based search, nearly 20,703 sugarcane ESTs were found from the EST database (dbEST) (11 December 2019) available at https://www.ncbi.nlm.nih.gov/genbank/dbest/dbest_summary. Now, for profiling of probably conserved miRNAs, the reference miRNAs and sugarcane ESTs have been submitted to BLASTn and BLASTx algorithms by eliminating the protein-coding and repetitive sequences.^30^ Hence, the putative candidate sugarcane miRNAs in FASTA format with non-coding features and up to four mismatches with the source miRNAs were isolated, maintained, and sent for further assessment.

### Sugarcane miRNAs stem-loop structures

To profile and describe novel conserved miRNAs in sugarcane, the primary technique applied is the creation of stem-loop secondary structures of initial probable candidate sequences.^5^ Hence, using a tool-like MFOLD (version 3.6), stem-loop structures were produced to determine secondary structure for the initial found potential sugarcane miRNA sequences.^31^ Likewise, all those potential candidate sequences that were initially unsuccessful in generating secondary structures have been withdrawn.

Later on, the choice was limited to only those potential candidate miRNA sequences which have stable secondary structures demonstrating mature sequences in the stem section, contain about 12 nucleotides in Watson-Crick or G/U base pairing with the opposing strand and show ≤ −10 Kcalmol^−1^ minimum free energy (MFE) were saved and put under physical scrutiny.

### Identification and filtration of miRNAs candidates from sugarcane ESTs

It is a vital step that removes all the false positive miRNAs from the candidate miRNAs. Hence, all potential candidate miRNAs resulting from sugarcane ESTs have characteristics such as creating a stable stem-loop secondary structure, non-coding in behavior, single-tone in natures, and maximum 4 mismatches with the reference miRNAs were physically analyzed to remove the sequences of large bulges, having higher MFEs and mature sequences not in the stem section. The further significant point is that each newly discovered sugarcane miRNA has an EST that identifies the organ in which it is expressed.

### Materials

Trizol reagent, Ethylene Diamine Tetra Acetate (EDTA), Glacial acetic acid, Liquid Nitrogen, Chloroform Isoamyl alcohol, Ethanol, Sodium acetate, DEPC treated water, cDNA synthesis kit, Taq DNA polymerase, PCR master mix, 100 base pair DNA ladder.

### RT-PCR validation

In light of the newly profiled sugarcane miRNAs, 14 miRNAs were randomly picked and exposed to an expressional analysis by RT-PCR (Reverse Transcription).^18,32^ Considering this, the Primer-3 algorithm (http://bioinfo.ut.ee/primer3-0.4.0.) was used to make stem-loop primers from the ESTs of 14 subjectively picked miRNAs (Table 1). With the use of Trizol reagent (Cat No: AM9738, Thermo Scientific), total RNA was successfully extracted from sugarcane leaves. Following that, cDNA was made utilizing the RevertAid™ First Strand cDNA Synthesis Kit (Cat No: K1622, Thermo Scientific) following the supplier’s protocol. A PCR mixture of 60 μl cDNA was used as a template for PCR amplification. Further adjustment of PCR should be like this: Preheat (activation) at 95°C for 5 min, denaturation at 95°C for 45 sec for 35 cycles, annealing at 60°C for 45 sec, extension at 72°C for 1 min and post cycling extension step at 72°C for 5 min. The findings for separating PCR products were obtained using a 1.5% (w/v) agarose gel with a 100-base pair DNA ladder.

**Table 1.** Denotes the 14 randomly picked sugarcane forward and reverse primers along with accession number, amplicon size, melting temperature, GC % and number of bases.

Sugarcane miRNAs	Accession	Primer (Forward and Reverse)	Amplicon size	Tm	GC%	Bases
sof-miR166a	CN607727	F- TCTGAGTGGAATGTTGTCTG *R*- AAAAGGCACAGTGGAGTAAT	301	55.01 54.84	45.00 40.00	20 20
sof-miR444a	CA107795	F- ATCGTCGTCTCAGATTGATG *R*- TTTGGAATGGATGGAGTACC	303	55.11 54.70	45.00 45.00	20 20
sof-miR827a	CA215078	F- GACCATCAGCAAACATGTTC *R*- GGAAGAGGAAGAGGAAGAAC	308	55.26 54.79	45.00 50.00	20 20
sof-miR1848b	CA107130	F- TAGCCCACATAACTAGGACA *R*- ACATTCTACATGACTCGCG	415	54.94 55.16	45.00 47.37	20 19
sof-miR1861n	CA300285	F- GCTTCGATGACTATGCCATT *R*- ACATGGTGAAGGTCCGTT	417	55.99 56.02	45.00 50.00	20 18
sof-miR2926b	CA197829	F- TAGTATGAGCTCCGACGG *R*- TATTACACAACGGGCTGAAC	330	55.18 55.78	55.56 45.00	18 20
sof-miR5025a	CA105537	F- CATATATATGTCCGGGTGCA *R*- ATCGTGTCACCTTAACATTT	335	54.48 52.82	45.00 35.00	20 20
sof-miR5075c	CA104318	F- GGGTGTTTGATGTTAAGAGC *R*- AATTTCACACTCACCAGCA	390	54.59 54.91	45.00 42.11	20 19
sof-miR5168a	CN607727	F- TCTGAGTGGAATGTTGTCTG *R*- AAAAGGCACAGTGGAGTAAT	296	55.01 54.84	45.00 40.00	20 20
sof-miR5564c	CN607542	F- CCTAAACTAAACCACCATTCC *R*- AAGTTTAAACAAAGCAGACCG	431	54.16 55.28	42.86 38.10	21 21
sof-miR5568a	CN608955	F- TGGTTGGATAAATTCTGCCA *R*- CCTTGTATTGCTGTGGAAAC	453	54.89 54.87	40.00 45.00	20 20
sof-miR6164b	CA129539	F- CACCCTCTGTCCCAAATTAT *R*- TGATCTGTGCAATCTTAGCT	317	54.69 54.45	45.00 40.00	20 20
sof-miR6192	CA119738	F- TATAGTGTAGCTTGGGACCA *R*- CTGAGGAGAGAAGAGACCC	302	54.94 55.53	45.00 57.89	20 19
sof-miR6225a	CA120709	F- GACCGTGTTTAGATCCCAAT *R*- TGGAAGTTTGAGGGGATCTA	281	55.14 55.12	45.00 45.00	20 20

### Phylogenetic and conservation analyses

In this, miR-166a phylogenetic analysis was initiated by comparing it to other monocotyledonous and dicotyledonous plant precursors connected to *Saccharum officinarum* (sof), *Oryza sativa* (osa), *Zea mays* (zma), *Nicotiana tabacum* (nta), *Populus trichocarpa* (ptc) and *Citrus sinensis* (csi) via a tool easily accessible at (https://www.ebi.ac.uk/Tools/msa/clustalo/). Similarly, another program called Interactive Tree of Life (iTOL) with a link (https://itol.embl.de/) was utilized to improve the tree. It was completed using the approach described by Baqi et al.^18^ But, for conservation analysis a tool named as web logo which can be accessed through the link (http://weblogo.berkeley.edu/logo.cgi, version 2.8) was used to conduct studies on the sequence logo generator for conservation analysis of several plant precursors like *Oryza sativa* (osa), *Citrus sinensis* (csi) and *Brachypodium distachyon* (bdi) to that of *Saccharum officinarum* (sof). A similar process was utilized for logo generation, as reported by Baloch et al. (2018) and Baqi et al. (2024c).^26,33^

### Targets prediction

For the prediction of possible targets of the newly found sugarcane miRNAs, psRNATarget: A Plant Small RNA Target Analysis Server (2017 Update) zhaolab.org, available at (http://www.zhaolab.org/psRNATarget/) was utilized.^18^ The sugarcane library (*Saccharum officinarum* (sugarcane), unigene, DFCI Gene Index (SOGI), version 3, released on 09-04-2010) was utilized as the preferred target library with the revised 2017 restructured parameters of psRNA Target as Seed region (2–13), Calculating UPE (No), Penalty for opening gap (2.0), # of mismatches allowed in the seed region (2), Penalty for extending gap (0.5), Allowing bulge on target (Yes), HSP size (19), Penalty for G:U pair (0.5), Weight for seed region (1.5), Penalty for other mismatches (1.0) and Max Expectation cutoff (5).^16^

## Results and discussion

### Sugarcane new potential miRNAs

To find out new exciting outcomes of various organisms, the extensively used strategy is comparative genomics-based research.^26,34^ In this research, 74 freshly conserved miRNAs were formed from sugarcane ESTs using a comparative genomics-based homology search. The 74 newly conserved miRNAs are associated with 41 miRNA families. They contain sof-miR166a, 166b, 166c, 390a, 444a, 482b, 827a, 827b, 1128, 1848a, 1848b, 1861n, 2094b, 2094c, 2098a, 2098b, 2118a, 2118b, 2120a, 2120b, 2120c, 2926a, 2926b, 5025a, 5025b, 5048a, 5048b, 5075a, 5075b, 5075c, 5075d, 5168a, 5168b, 5181a, 5181b, 5281a, 5281b, 5281c, 5337a, 5337b, 5337c, 5337d, 5502a, 5502b, 5564c, 5568a, 5568b, 5568c, 5568d, 5831, 6164b, 6192, 6220a, 6220b, 6220c, 6220d, 6225a, 6225b, 6235, 6249a, 6249b, 6332a, 6332b, 6332c, 6332d, 7768a, 7768b, 7768c, 7768d, 8005a, 8005b, 8005c, 8005d and 9780 (Table S1).

Furthermore, it is proved that these new 74 miRNAs of sugarcane have been reported for the first time and have not been published earlier. Accordingly, these 74 novel miRNAs have been developed with the support of source miRNAs of *A*. *officinalis* (1%), *A*. *tauschii* (5%), *A*. *thaliana* (3%), *B*. *distachyon* (10%), *C*. *sinensis* (1%), *H*. *vulgare* (3%), *N*. *tabacum* (1%), *O*. *sativa* (35%), *S*. *bicolor* (24%), *S*. *tuberosum* (5%), *T*. *aestivum* (4%), and *Z*. *mays* (8%). In this context, to meet the empirical criteria A, B, and D for the production and expression of miRNAs as outlined by Ambros et al. (2003), all recently identified conserved miRNAs in sugarcane have been regarded as confirmed candidates. According to Ambros et al. (2003), the criterion D is only sufficient for demonstrating the presence of new miRNAs based on homologous sequences across different plant species.^35^

### Sugarcane miRNAs characterization

**Figure 1. (continued).**
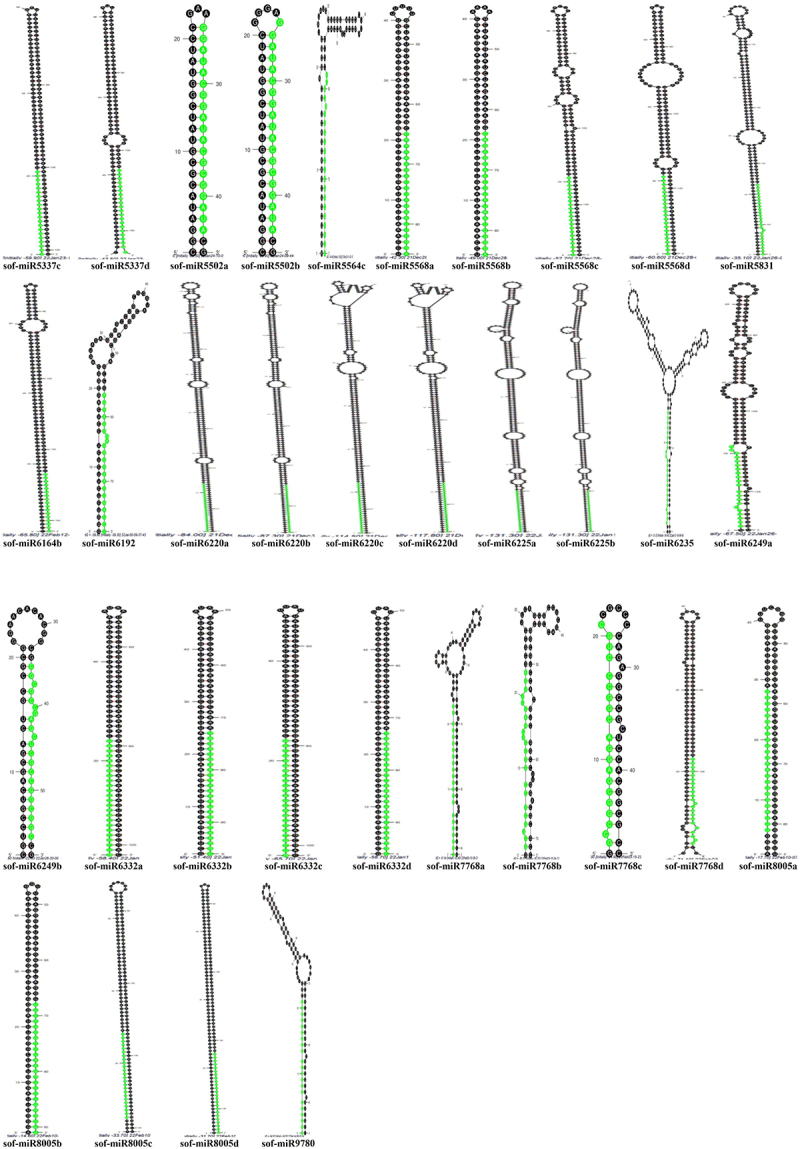

The newly reported sugarcane miRNAs have been classified and described in respect of pre-miRNAs length, MFE of pre-miRNAs, mature miRNA sequences with mismatches, number of mismatches, mature sequence length, ESTs, strand orientation, mature sequences arm, GC percentage and organ of expression (Table S1). Subsequently, the mature sequences of newly conserved sugarcane miRNAs are observed in the stem parts of the stem-loop structures (Figure 1).
**Figure 1.**
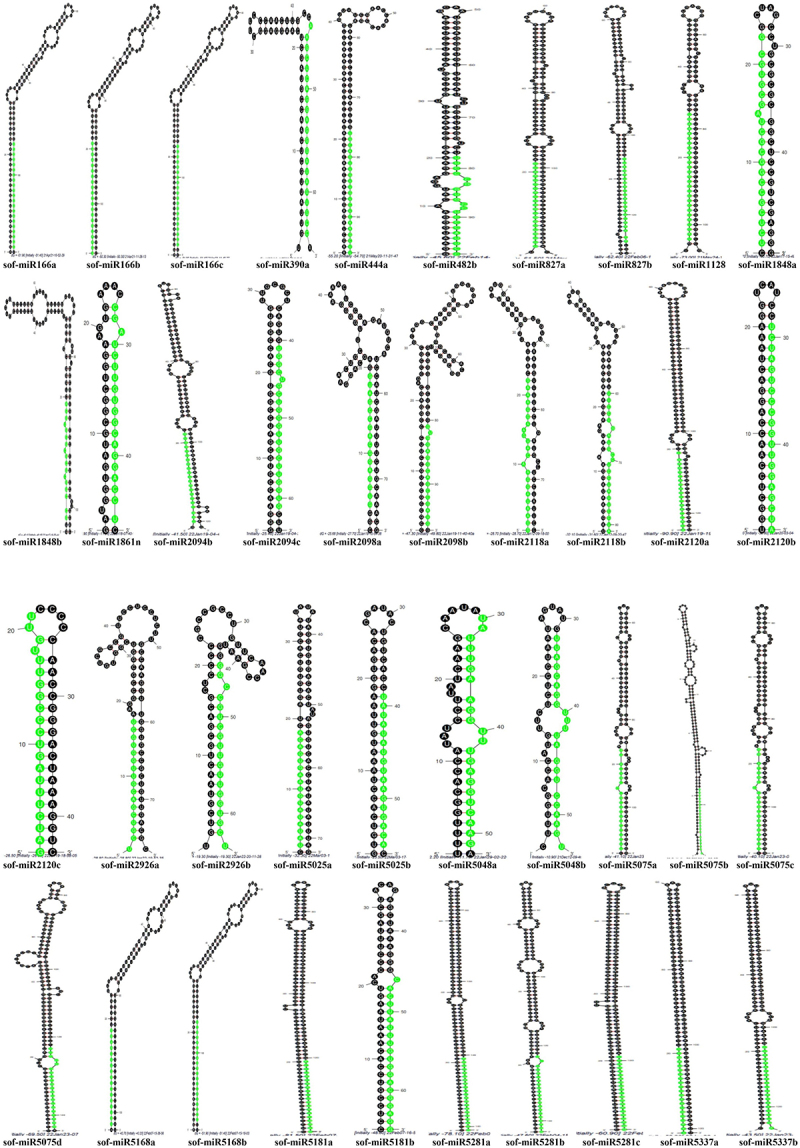
The secondary structures of the recently discovered sugarcane miRNAs. The secondary structure of the sugarcane pre-miRNAs was produced by the Mfold method. The mature miRNAs (in green) in the stem region of the stem loop structures are readily seen in the above structures.

**Figure 2.**
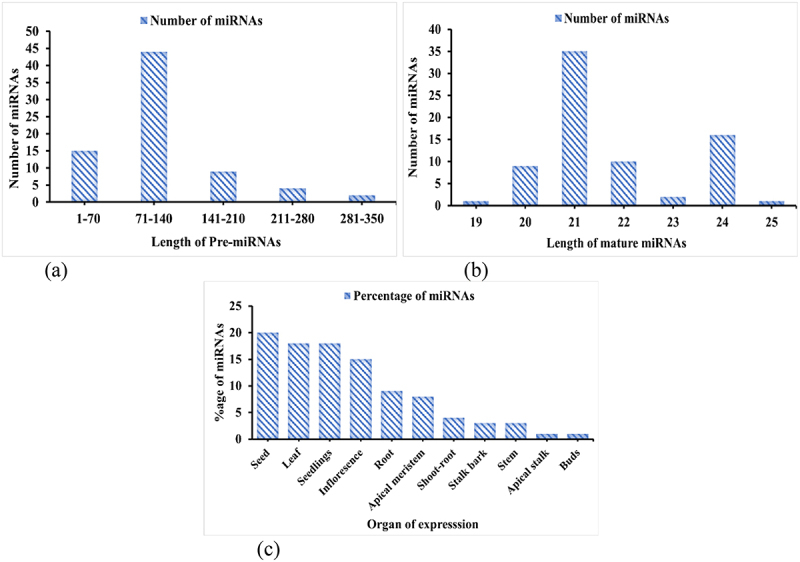
The distributions identified in sugarcane ESTs (a) length of precursor miRNAs (b) length of mature miRNAs (c) organ of expression.

It is shown that the stem-loop structures of the anticipated miRNAs include nearly 11–21 nucleotides that are engaged in Watson – Crick or G/U base pairings between the mature miRNA and the opposing arms (pre-miRNAs) in the stem portion. Also, the ancestors of hairpins lack considerable interior loops or bulges. Research has revealed corresponding miRNAs results in numerous plants and animals.^36^ Generally, sugarcane pre-miRNAs vary in length from 43 to 303 nt, averaging 113 nt. Considerably, pre-miRNAs with lengths between 1 and 70 nt (15 out of 74) make up a sizable portion of the pre-miRNA, accounting for 20%. Likewise, from 71 to 140 nt (44 out of 74) 59%, 141–210 nt (9 out of 74) 12%, 211–280 nt (4 out of 74) 6%, and 281–350 nt (2 out of 74) 3% (Figure 2a).

Furthermore, this work has highlighted that the MFE of the recently discovered sugarcane pre-miRNAs ranges from −131.8 to −10.9 kcal mol^−1^ with an average of −49.29 kcal mol^−1^. Following class boundaries −150 to −120 kcal mol^−1^ (2) formed 3% of the overall pre-miRNA, from −119 to −90 (3) 4%, from −89 to −60 kcal mol^−1^ (17) 23%, from −59 to −30 kcal mol^−1^ (35) 47% and from −29 to −0.0 kcal mol^−1^ (17) 23% of all the pre-miRNAs. The findings about the reported MFEs of pre-miRNAs that were previously addressed were supported by other researchers working with various organisms.^9,13,36^

As mentioned above, the study found that the total number of mismatches found in the anticipated sugarcane mature miRNAs and their source sequences ranged from 0 to 4, with an average of 3 mismatches. Henceforth, 4 mismatches (38 miRNAs out of 74) are sought in 51% of whole miRNAs, 3 mismatches (10 miRNAs out of 74) with 14%, 2 mismatches (14 miRNAs out of 74) with 19%, 1 mismatch (6 miRNAs out of 74) with 8% and accurately matched were 8% (6 miRNAs out of 74). The outcomes of sugarcane miRNA mismatches, which vary from 0 to 4, are comparable to those for the other plant and animal species that have already been mentioned.^18,36^

Accordingly, the mature lengths of sugarcane miRNAs were discovered to range between 19 and 25 nt, with an average length of 22 nt. Assuming the class boundaries, the lengths of the mature sequences vary from shortest to longest as follows: 19 nucleotides (nt) account for 1 out of 74 sequences, representing 1% of the total; 20 nt comprise 9 out of 74 sequences, or 12%; 21 nt make up 35 out of 74 sequences, equating to 47%; 22 nt consist of 10 out of 74 sequences, which is 14%; 23 nt include 2 out of 74 sequences, corresponding to 3%; 24 nt represent 16 out of 74 sequences, totaling 22%; and 25 nt contain 1 out of 74 sequences, also accounting for 1% (Figure 2b). As a result, the length range of sugarcane mature sequences is similar to the other known plant miRNAs.^16^ This study found 38 of the 74 newly examined miRNAs, or 51% of all miRNAs, in the sense strand. Contrarily, 36 of the 74 or 49% of the entire miRNAs were formed in an anti-sense strand orientation. In addition, 32 out of 74 miRNAs, or 43% of all mature sequences, were seen on the 5‘arm of secondary structures, whereas 42 out of 74 miRNAs were found to form 57% on the 3’ arm. Considering nucleotide sequence, the essential measure of characterization is the GC percentage. Therefore, the newly predicted sugarcane miRNAs were found to have a GC percentage ranging from 24% to 91%, averaging 50%. Based on the class boundaries, the distribution of GC content (GC%) is as follows: 0% to 30% comprises 7 out of 74 sequences, representing 9%; 31% to 60% includes 48 out of 74 sequences, equating to 65%; 61% to 90% accounts for 18 out of 74 sequences, which is 25%; and 91% to 120% contains 1 out of 74 sequences, making up 1% of the total.

Similarly, the organ of expression of the freshly explored sugarcane miRNAs has also been computed for their ESTs. The majority of miRNAs are found in the seeds (15 out of 74), which make up 20% of the total and followed by leaf 18%, seedlings 18%, inflorescence 15%, root 9%, apical meristem 8%, shoot-root 4%, stalk bark 3%, stem 3%, apical stalk 1% and buds 1% (Figure 2c). At the beginning of the development and regulation of enhanced plant organs, the expression of sugarcane miRNAs at the organ level plays essential roles. Data from other plant species that have been previously published are consistent with the various organ-based expression of miRNAs that have been described using comparative genomics techniques.^16–19^

### Amplification and validation of sugarcane miRNAs

**Figure 3.**
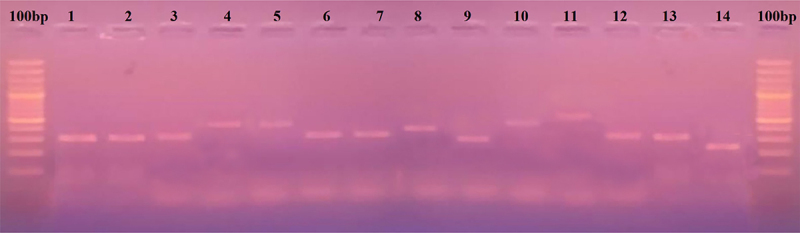
Sugarcane miRNAs RT-PCR expressional validation. A 100 base pair ladder and fourteen sugarcane miRnas; 1 (sof-miR166a), 2 (sof-miR444a), 3 (sof-miR827a), 4 (sof-miR1848b), 5 (sof-miR1861n), 6 (sof-miR2926b), 7 (sof-miR5025a), 8 (sof-miR5075c), 9 (sof-miR5168a), 10 (sof-miR5564c), 11 (sof-miR5568a), 12 (sof-miR6164b), 13 (sof-miR6192) and 14 (sof-miR6225a) were chosen and analyzed for expression using RT-PCR for experimental validation. End result of every sample was isolated using an agarose gel with a 1.5% (w/v) agarose content that contains a DNA ladder (100 base pair).

The significant method employed is the RT-PCR for the experimental validation of the freshly profiled sugarcane miRNAs. Fourteen sugarcane miRNAs and 100 base pair ladders were used for amplification^32^ in RT-PCR expressional experiment. The arrangement will be like: 1 (sof-miR166a), 2 (sof-miR444a), 3 (sof-miR827a), 4 (sof-miR1848b), 5 (sof-miR1861n), 6 (sof-miR2926b), 7 (sof-miR5025a), 8 (sof-miR5075c), 9 (sof-miR5168a), 10 (sof-miR5564c), 11 (sof-miR5568a), 12 (sof-miR6164b), 13 (sof-miR6192) and 14 (sof-miR6225a) (Table 1). Fourteen sugarcane miRNAs have all been appropriately validated by RT-PCR, and the findings are displayed (Figure 3). Hence, the 14 products were examined using an agarose gel with a 1.5% concentration and a 100-base pair DNA ladder. Researchers investigating different plant varieties have used these results.^36^

### Phylogenetic and conservation studies of sugarcane miRNAs

**Figure 4.**
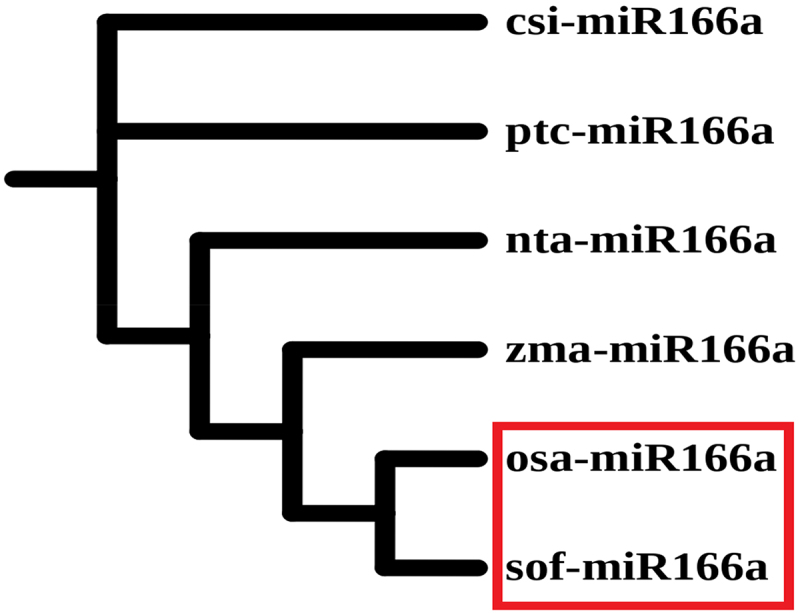
Sugarcane miRNA and their phylogenetic analysis. When compared to *O*. *sativa*, *Z*. *mays*, *N*. *tabacum*, *P*. *trichocarpa* and *C*. *sinensis*, a phylogenetic tree revealed that *O*. *sativa* is more closely related to *S*. *officinarum* (sof). Red box was used to draw attention to the nearby plant species.

**Figure 5.**
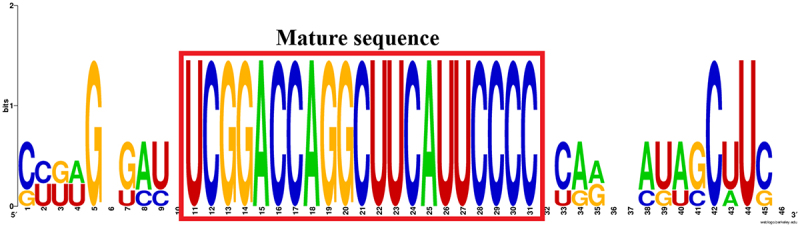
Conservation analysis of the miRNA in sugarcane. Mature miRNA sequences and their conserved nature are shown in the red boxed area that has been highlighted.

The phylogenetic tree and conservation studies for sugarcane miRNAs are created and presented in Figures 5 and 6, respectively. Sugarcane and a grass species named rice (*Oryza sativa*) are closely related, as seen by the red highlighted box in Figure 4. According to phylogenetic analyses of sugarcane miRNAs, the sof-miRNA166a is more closely related to *O*. *sativa* (osa) than to *Z*. *mays* (zma), *N*. *tabacum* (nta), *P*. *trichocarpa* (ptc) and *C*. *sinensis* (csi). In agreement with conservation studies of the pre-miRNA 166a shown in Figure 6, the red highlighted frame exhibits the conserved regions of matures linked to other plants such as *O*. *sativa*, *C*. *sinensis*, and *B*. *distachyon*. Similar outcomes have already been proposed by specialists from many professions.^16–18^

### Evaluation of sugarcane miRNAs primary targets

Estimating the targets is imperative to fully understand and characterize the freshly found sugarcane miRNAs. Mostly 11,453 target genes have been noted for the newly identified 74 freshly conserved sugarcane miRNAs with the help of a highly complex technique, as explained above. According to Baqi (2023), a single miRNA can target a group of proteins.^18^ An extensive summary of sugarcane mature miRNAs and their predicted targets has been provided (Table S2; Figure 6). The findings demonstrate that a single miRNA can target many sugarcane genes. There were 11,453 essential targeted proteins in total, of which 50 significant targets for different proteins were chosen, and they were divided into distinct classes based on their functional roles. In addition to these interrelated functions are those of structural proteins, transcriptional regulators, metabolic processes, cell signaling proteins, and transporters.

**Figure 7.**
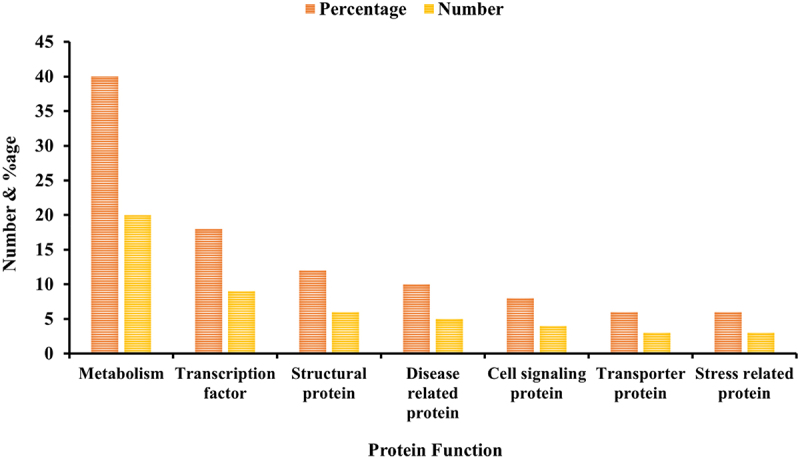
Important proteins target graph. This shows the number and percentage of different sugarcane miRNAs targeted proteins.

The part of metabolic functions contained most of the targeted proteins found. They constituted 20 out of 50, or 40% of the targeted proteins for the observed miRNAs. Various attained metabolism classes include UDP-glycosyltransferase-like protein, methionine aminopeptidase, DNA-directed RNA polymerase, adenine phosphoribosyltransferase, xyloglucan endotransglycosylase, N-acetylmuramoyl-L-alanine amidase, amino acid permease 6, cinnamoyl ester hydrolase, ATP citrate lyase alpha subunit, etc. The primary role of these targeted proteins is metabolism, which involves them in various metabolic processes like the sugarcane plant cell cycle and meristem formation.^27^

The second class of proteins comprises the targeted proteins, including those that fall under transcription factors. They are a part of every plant and are necessary for the development of plants.^27^ Per the information gathered, nine out of fifty transcription factors, or 18% of the targets, were noticed. Among the factors that were acquired include small Ras-related GTP-binding protein, harpin-induced protein 1 containing protein, nuclear transcription factor Y subunit B-4, RNA binding protein, extracellular calcium-sensing receptor, etc. Particularly, the sof-miR6249b helps in the expression of zinc finger, C_2_H_2_ type protein.

The targeted protein obtained in the third class was the structural protein. These proteins can encourage the slackening and lengthening plant cell walls by interrupting non-covalent connections between cellulose microfibrils and matrix glucans. Moreover, they work on developing the root, stalk, leaf, flower, etc. Accordingly, these proteins contain vegetative cell wall protein, beta-expansin 7 precursor, jasmonate induced protein, histone-like protein, etc. In this class, 6 of the 50 structural proteins were achieved, giving 12% of the targeted proteins. Numerous researchers have reported multiple structurally targeted proteins in numerous plants using this method.^27,37^

The target proteins found for the fourth class are disease-related. They comprised five out of fifty, or 10% of all known miRNAs that target proteins. In fact, the sof-miR7768b regulate the pathogenesis-related protein. Researchers using the same method have previously discovered that various plants’ miRNAs target distinct disease-related proteins differently.^27^

Analysis indicates that cell signaling proteins, which are involved in signal transmission in a cell, constitute the fifth category of targeted proteins. Consequently, 4 out of 50 signaling proteins were discovered, representing 8% of all targets. The targeted proteins that were effectively obtained involve nucleosome/chromatin assembly factor C, protein dehydration-induced 19, AT-rich interactive domain-containing protein 3A and splicing factor, and arginine/serine-rich 2. Researchers have presented numerous cellular signaling targets in various plants.^36^

In the next class, several targeted transporters and stress-related proteins by sugarcane miRNAs have been given. According to the observed results, the examples of transport proteins include Antheraea pernyi fibroin, transporter-like protein, and voltage-dependent anion channel protein 2. The sof-miR8005a regulate the voltage-dependent anion. In contrast, the stress-related proteins comprise heat repeat family protein, actin-depolymerizing factor 5, and fasciclin-like protein FLA13. The heat repeat protein is governed by sof-miR5025. It was noted that both these classes would include 3 out of 50 transport proteins, or 6% of the overall targeted proteins. There have been reports of many targeted transport and stress-related proteins created using the same method in various plants.^36^

## Conclusion

In summary, this research is the first to reveal the presence of 74 unique potential sugarcane miRNAs, members of 41 distinct miRNA families. New and advanced bioinformatics tools have been used to anticipate and evaluate these miRNAs. Moreover, fourteen miRNAs were randomly selected to act as primer templates, and RT-PCR validated the primers. Considering the primary targets, the newly characterized miRNAs in sugarcane exhibited 11,453 various protein targets using the psRNA Target method. It led to the success of 50 specifically targeted proteins that were then integrated into the primary targets in multiple activities like metabolism, cell signaling, transportation, structure, and transcription. Moreover, sof-miR-7768a controls arginine/serine, a splicing factor that aids in cell signaling proteins, while sof-miR-2926b controls the extracellular calcium-sensing receptor, a transcription factor supporter. So, these findings showed that sugarcane miRNAs target a wide range of associated genes and have the power to influence the environment and system to increase the production of the sugarcane plant. Some of the limitations of the current study like the identification of novel miRNAs requires more experimental validation, miRBase has a large degree of non-experimentally validated miRNAs that were annotated purely based on the existence of small RNA fragments and the non-consumption of folding algorithms like INFERNAL used for conservation across species due to financial and time restriction issues. Furthermore, the non-validation of function of miRNAs like processing and AGO-binding, repression of a reporter, dependency on miRNA biogenesis genes such as DCL1, the non-availability of qRT-PCR and sequencing data, and the limitation of resources and funding and scarcity of data are the limitations of the study.

## Supplementary Material

Table_S2_Computational clean.docx

Table_S1_Computational clean.docx

## Data Availability

All data generated or analyzed during this study are included in this published article.
