# Supplementary material for: Computational identification and experimental validation of novel Saccharum officinarum microRNAs along with their targets through RT-PCR approach
Source: Plant Signal Behav. 2025 Jan 28;20(1):2452334. doi: 10.1080/15592324.2025.2452334 (PMC11776470; doi:10.1080/15592324.2025.2452334)
Supplement: Table_S2_Computational clean.docx [file KPSB_A_2452334_SM6991.docx]

**Table S2.** Evaluation of possible sugar cane targets predicted by psRNATarget in term of miRNAs family number, targets accession, targets description, functions and their alignment.

| ***Saccharum officinarum* miRNAs** | **Target Accession** | **Target Description** | **Function** | **Alignment** |
| --- | --- | --- | --- | --- |
| sof-miR166a | CA150682 | Small Ras-related GTP-binding protein | Transcription factor | miRNA 21 CCCCUUACUUCGGACCAGGCU 1  ::::::: .:::::::::  Target 743 CAAGAAUGAA-UCUGGUCCGA 762 |
| sof-miR166b | TC137338 | UDP-glycosyltransferase-like protein | Metabolism | miRNA 21 GGAACUUGGUCUGUUGUAAGG 1  :.::::.::.:::. :::: :  Target 14 CUUUGAGCCGGACGCCAUUAC 34 |
| sof-miR166c | TC121002 | Harpin-induced protein 1 containing protein | Transcription factor | miRNA 20 CCCUUACUUCGGACCAGGCU 1  : ..:::.:.::::::::  Target 808 GCCGGUGAGGUCUGGUCCGU 827 |
| sof-miR390a | CA112540 | Nucleosome/chromatin assembly factor C | Cell signaling protein | miRNA 21 CCGUGAUAGGGAGGACUCGAA 1  ::: .:::::::. ::::::  Target 526 GGCUUUAUCCCUUACGAGCUU 546 |
| sof-miR444a | CF572862 | Methionine aminopeptidase | Metabolism | miRNA 21 UUCGAACUCUGUUGUUGACGU 1  .:::: :::.:::::: :::  Target 360 GAGCUGGAGGCAACAAAUGCU 380 |
| sof-miR482b | CA256896 | Protein dehydration-induced 19 | Cell signaling protein | miRNA 22 CCUUACCCUCCGUGGCCCUUCU 1  : :::::::::::::::::  Target 334 GUCUUGGGAGGCACCGGGAAGC 355 |
| sof-miR827a | GE325037 | Bacteriodes thetaiotaomicron symbiotic chitinase | Disease related protein | miRNA 21 CCAAUUUACUGGUGGUUGUUU 1  : : :.::::::.::::::.  Target 545 GCAUUAGUGACCAUCAACAAG 565 |
| sof-miR827b | CA183620 | Nuclear transcription factor Y subunit B-4 | Transcription factor | miRNA 21 ACAAACGACUACCAGUAGAUU 1  :::::.:::::: :: ::::  Target 607 AGUUUGUUGAUGG-CACCUAA 626 |
| sof-miR1848a | CA203355 | Vegetative cell wall protein | Structural protein | miRNA 21 GCGUGCGAUCGCGGCCGCUCU 1  ::::: .::::::::::::  Target 201 AGCACGGCGGCGCCGGCGAGA 221 |
| sof-miR1848b | TC139701 | RNA binding protein | Transcription factor | miRNA 21 ACGUGCGCGCGCGGCCGCUCC 1  :::: :::::::::::::::  Target 227 GGCAC-CGCGCGCCGGCGAGG 246 |
| sof-miR1861n | CA102550 | DNA-directed RNA polymerase | Metabolism | miRNA 22 GAGUCCAGGACGGUGUUCUAGC 1  ::::: :.:::::::.::::  Target 483 GUCAGGACUUGCCACAGGAUCA 504 |
| sof-miR2094b | TC149872 | AT-rich interactive domain-containing protein 3A | Cell signaling protein | miRNA 21 GUGGUUCGUUGGGUCGUCGGU 1  ::::::::::::: ::::.:  Target 344 AACCAAGCAACCCAACAGCUA 364 |
| sof-miR2094c | TC133465 | Adenine phosphoribosyltransferase | Metabolism | miRNA 21 GUGGUUCGUUGGGUCGUCGGU 1  ::::::::.:::: ::::.:  Target 402 AACCAAGCAGCCCAACAGCUA 422 |
| sof-miR2098a | CA293977 | Xyloglucan endotransglycosylase | Metabolism | miRNA 20 GUAGCCGACGGAGGUGCCCU 1  ::: :::::: :::.:::::  Target 600 CAUGGGCUGCGUCCGCGGGA 619 |
| sof-miR2098b | TC150814 | Beta-expansin 7 precursor | Structural Protein | miRNA 20 GUAGCCGACGGAGGUGCCCU 1  ::.::.:::.::::.:: :  Target 1243 AAUUGGUUGCUUCCAUGGAA 1262 |
| sof-miR2118a | DV730336 | Jasmonate induced protein | Structural Protein | miRNA 22 GAAAGGAAGUACAAGGAUAAGG 1  . :::::: :::::::::::.  Target 114 UGUUCCUUGCUGUUCCUAUUCU 135 |
| sof-miR2118b | TC125681 | BRI1-KD interacting protein 135 | Disease related protein | miRNA 22 AUCCUUAUCCUCCGUAGUCCUU 1  ::. :::::::::::::::  Target 575 AUAGAGCAGGAGGCAUCAGGAA 596 |
| sof-miR2120a | CA153971 | Histone-like protein | Structural protein | miRNA 19 CAAAUCAGAGCCAACCACA 1  ::: . ::::::::::::  Target 697 GUUCGUUCUCGGUUGGUGC 715 |
| sof-miR2120b | CA118554 | N-acetylmuramoyl-L-alanine amidase | Metabolism | miRNA 20 AUCGAGGUUGGCCCUGAUCU 1  :: :.:.::.:::::::::  Target 304 AAGAUUCGACUGGGACUAGA 323 |
| sof-miR2926a | TC124856 | Stress-induced transcription factor NAC1 | Transcription factor | miRNA 20 UCGUGGUUGCAGCUGCUGGA 1  .:::.:::: ::::::::::  Target 428 GGCAUCAACCUCGACGACCU 447 |
| sof-miR2926b | CA295286 | Extracellular calcium sensing receptor | Transcription factor | miRNA 20 UCGUGGUUGUUGCUGCCGGA 1  :::.:::::.::::::::::  Target 435 AGCGCCAACGACGACGGCCU 454 |
| sof-miR5025a | TC145756 | HEAT repeat family protein | Stress related protein | miRNA 21 ACAGUGAAUGUAUAUAUGUCA 1  :.::::..::::.::::::  Target 1050 AUUUACUUGUAUAUGUACAGU 1070 |
| sof-miR5025b | CA231585 | Amino acid permease 6 | Metabolism | miRNA 21 ACAGUGAAUGUAUAUAUGUCA 1  :: ..: ::::::::::::::  Target 433 UGAUGCAUACAUAUAUACAGU 453 |
| sof-miR5048a | TC150305 | Wound responsive protein | Disease related protein | miRNA 22 GAACUGGAGUUUGGAAGUUUAU 1  .::::::::::.:: :::.::.  Target 300 UUUGACCUCAAGCCAUCAGAUG 321 |
| sof-miR5048b | CA122621 | 40S ribosomal protein S12 | Structure protein | miRNA 22 UGAACCUGGAUUUUGGACGAUU 1  .:::: :..:....:.:::::.  Target 548 GCUUGCAUUUGGGGCUUGCUAG 569 |
| sof-miR5075b | CA110857 | Actin-depolymerizing factor 5 | Stress related protein | miRNA 21 GGCCUGCCUGCGCUGCCUCUU 1  :::.:::::.:: ::::.:  Target 125 GGGGAUGGACGUGAAGGAGGA 145 |
| sof-miR5075c | CA288750 | Thioredoxin h2 protein | Disease related protein | miRNA 21 GGACUGCCUGCGCUGCCUCUU 1  ::.:::.::::.:::::..  Target 2 AAUGGCGGGCGCGGCGGAGGG 22 |
| sof-miR5181a | CA259836 | Cinnamoyl ester hydrolase | Metabolism | miRNA 21 GAGGCAAGGUUUAAUAUUCAC 1  :::.::::::::::::::::  Target 598 CUCUGUUCCAAAUUAUAAGUU 618 |
| sof-miR5181b | BQ534080 | Plasma membrane Ca2+-ATPase | Metabolism | miRNA 20 GGAGGCAGGUGUUUAUUCAC 1  . :..::.::::::: .:::  Target 269 UGUUUGUUCACAAAUUGGUG 288 |
| sof-miR5281a | CA215868 | ATP citrate lyase alpha subunit | Metabolism | miRNA 21 GGAGGCAAGAUUUAAUAUUCA 1  .:::::::: ::::::::::  Target 47 UCUCCGUUCCAAAUUAUAAGC 67 |
| sof-miR5281b | TC113248 | Ubiquitin carboxyl-terminal hydrolase 1 | Metabolism | miRNA 21 GGAGGCAAGGUUUAAUAUUCU 1  .:::::::::::.::::::::  Target 711 UCUCCGUUCCAAGUUAUAAGA 731 |
| sof-miR5337a  sof-miR5337c | CA099662 | Glycine-rich RNA-binding protein | Transcription factor | miRNA 21 UCGAUCUUGCUGAAUGUUAAA 1  ::::.:: :::::.::: :  Target 508 CACUAGGACUACUUAUAAUGU 528 |
| sof-miR5337b | CA220994 | Endoglucanase Y | Metabolism | miRNA 21 AGUUUAACAUUCAGCAAGAUC 1  ::::::::::: :::::.:.:  Target 28 UCAAAUUGUAAUUCGUUUUGG 48 |
| sof-miR5502a | CA134984 | Glycosyltransferase | Metabolism | miRNA 23 CAUAGGCGCAUAGGCAUAGGCAU 1  :.:.::::..::::..:::  Target 313 ACUGCUGUGUAUUUGUAUUUGUA 335 |
| sof-miR5502b | CA260828 | Glyceraldehyde-3-phosphate dehydrogenase | Metabolism | miRNA 19 GGCGCAUAGGCAUAGGCAU 1  ::.:::: :::.::::::  Target 1 GCGUGUAUACGUGUCCGUA 19 |
| sof-miR5564c | TC145789 | Histone H2A | Structural protein | miRNA 21 UUAAGCGGUUUGUCGAGCGCA 1  .:::.::::::..:::. ::  Target 672 GAUUUGCCAAAUGGCUUUCGA 692 |
| sof-miR5568a | CA092490 | Antheraea pernyi fibroin | Transporter | miRNA 21 AGGUUUAAUAUUCAGAAAAAC 1  .::::::::::::::::::  Target 557 CUCAAAUUAUAAGUCUUUUUU 577 |
| sof-miR5568c | CA293262 | Cytochrome c oxidase subunit I | Metabolism | miRNA 21 AUUGAAUGUUAAACCUUACCU 1  :.:::::::::::::: ::::  Target 2 UGACUUACAAUUUGGACUGGA 22 |
| sof-miR6164b | TC152154 | Chlorophyll a/b binding protein precursor | Transcription factor | miRNA 21 GGAGGCAAAGUUAAAUACACU 1  ..::.: :::.:::.:::::  Target 871 UUUCUGGAUCAGUUUGUGUGA 891 |
| sof-miR6192 | TC135319 | Salt-induced AAA-Type ATPase | Metabolism | miRNA 19 AGGGAAGGGGAGGGAGGAU 1  :::.::::.:::::::::  Target 1 UCCUUUCCUCUCCCUCCUC 19 |
| sof-miR6220d | CA122622 | Glutathione peroxidase | Metabolism | miRNA 24 GGAGGUAGGGUUUAAUAUUCCGUA 1  .: ::::.:::.:::. :.:::.:  Target356 UCACCAUUCCAGAUUGAAGGGCGU 379 |
| sof-miR6225b | TC131103 | Thylakoid-bound ascorbate peroxidase | Metabolism | miRNA 24 UUGAUCCGAGUUUUCUAAGCAGAG 1  :::::::::::::::::::::::.  Target752 AACUAGGCUCAAAAGAUUCGUCUU 775 |
| sof-miR6249b | CA160083 | Zinc finger, C2H2 type family protein, expressed | Transcription factor | miRNA 20 CGGCGGGCGCUCGGCAGCGC 1  ::::::::::::::::::::  Target 422 GCCGCCCGCGAGCCGUCGCG 441 |
| sof-miR6332a | CA215868 | ATP citrate lyase alpha subunit | Metabolism | miRNA 24 UACAAGGUUUUUUCAGUUUCGCUG 1  ::: :::::::::::::::::::  Target146 AUGAACCAAAAAAGUCAAAGCGAC 169 |
| sof-miR6332d | CA140776 | 3-ketoacyl-CoA synthase 11 | Metabolism | miRNA 24 CGAAACUGAAAAAACCUUGUAGGU 1  :::::: : :::::::::::::  Target242 CCUUUGAAAUCUUUGGAACAUCCA 265 |
| sof-miR7768a | CA147054 | Splicing factor, arginine/serine-rich 2 | Cell signaling protein | miRNA 22 GAGGGGCAGCCCCUGCCGCGGC 1  ::.::: :::::.::::: ::  Target 199 GUCUCCGGCGGGGGCGGCGACG 220 |
| sof-miR7768b | CA111480 | Pathogenesis-related protein 2 | Disease related protein | miRNA 22 AGUUCCAGCUCCGGCCGCGGCG 1  .::: :::::::::::::::  Target 412 CGGAGGGCGAGGCCGGCGCCGC 433 |
| sof-miR7768c | CA079472 | Transporter-like protein | Transporter | miRNA 20 CGUCCCGGCAGGAGCGGCCG 1  :::: ::::::.:::::::  Target 316 GCAGCGCCGUCUUCGCCGGG 335 |
| sof-miR8005a | CA126418 | Voltage-dependent anion channel protein 2 | Transporter | miRNA 24 UUUGGGAUUUGGAAUUUGGGAUUU 1  ::::..:::::::.::::.:.  Target630 UCUCCCUGGACCUUAAGCCCUGAG 653 |
| sof-miR9780 | CA268734 | Fasciclin-like protein FLA13 | Stress related protein | miRNA 21 CGGCGCACGUCGCGGCUGCGG 1  : :::: ::.:::::::::::  Target 339 GGCGCGGGCGGCGCCGACGCC 359 |
