# Supplementary material for: Computational identification and experimental validation of novel Saccharum officinarum microRNAs along with their targets through RT-PCR approach
Source: Plant Signal Behav. 2025 Jan 28;20(1):2452334. doi: 10.1080/15592324.2025.2452334 (PMC11776470; doi:10.1080/15592324.2025.2452334)
Supplement: Table_S1_Computational clean.docx [file KPSB_A_2452334_SM6990.docx]

**Table S1.** Description of newly profiled sugarcane preserved miRNAs. The sugarcane projected miRNAs are categorized in respect of reference miRNAs, precursor miRNA length (PL), minimum free energy (MFE), mature sequences (MS), number of mismatches (denoted in bold and red) NM, mature sequence length (ML), source EST (SE), mature sequence arm (MSA), GC percentage (GC%), strand orientation (SO) and organ of expression (OE).

| ***Saccharum officinarum* miRNAs** | **Source miRNAs** | **PL** | **MFE** | **MS** | **NM** | **ML** | **SE** | **MSA** | **GC%** | **SO** | **OE** |
| --- | --- | --- | --- | --- | --- | --- | --- | --- | --- | --- | --- |
| sof-miR166a | zma-miR166a | 115 | -51.90 | UCGGACCAGGCUUCAUUCCCC | 0 | 21 | CN607727 | 3′ | 62 | + | Leaf |
| sof-miR166b | zma-miR166b | 109 | -50.30 | GGAAUGUUGUCUGGUUCAAGG | 0 | 21 | CN607727 | 5′ | 48 | + | Leaf |
| sof-miR166c | zma-miR166b | 115 | -51.90 | UCGGACCAGGCUUCAUUCCC | 0 | 20 | CN607727 | 3′ | 60 | + | Leaf |
| sof-miR390a | zma-miR390a | 65 | -14.80 | AAGCUCAGGAGGGAUAG**U**GCC | 1 | 21 | CA174685 | 3′ | 57 | - | Apical stalk |
| sof-miR444a | zma-miR444a | 96 | -55.20 | UGCAGUUGUUGUCUCAAGCUU | 0 | 21 | CA107795 | 3′ | 43 | + | Seedlings |
| sof-miR482b | csi-miR482 | 97 | -45.60 | **UC**UUCCC**GG**UGCCUCCCAUUCC | 4 | 22 | CA250529 | 3′ | 64 | + | Inflorescence |
| sof-miR827a | zma-miR827 | 120 | -61.50 | UUUGUUGGUGGUCAUUUAACC | 0 | 21 | CA215078 | 5′ | 38 | + | Seedlings |
| sof-miR827b | aof-miR827 | 129 | -62.90 | UUAGAUGACCAUCA**G**CAAACA | 1 | 21 | CA215078 | 3′ | 38 | + | Seedlings |
| sof-miR1128 | tae-miR1128 | 105 | -73.00 | UACUACUCCCUCCGUCC**GAAA** | 4 | 21 | CA283628 | 5′ | 52 | + | Seeds |
| sof-miR1848a | osa-miR1848 | 50 | -30.70 | **U**CUCGCCGGCGC**UA**GCGUGC**G** | 4 | 21 | CA082279 | 5′ | 76 | + | Apical meristem |
| sof-miR1848b | osa-miR1848 | 104 | -40.10 | **CCUC**GCCGGCGCGCGCGUGCA | 4 | 21 | CA107130 | 5′ | 86 | - | Seedlings |
| sof-miR1861n | osa-miR1861n | 46 | -11.90 | CGAUCUUGUGGCAGGA**C**CU**GAG** | 4 | 22 | CA300285 | 3′ | 59 | + | Leaf |
| sof-miR2094b | osa-miR2094 | 121 | -41.50 | UGGCUGCU**G**GG**U**U**G**CU**U**GGUG | 4 | 21 | CA076737 | 5′ | 62 | - | Apical meristem |
| sof-miR2094c | osa-miR2094 | 64 | -25.90 | UGGCUGCU**G**GG**U**U**G**CU**U**GGUG | 4 | 21 | CA076737 | 3′ | 62 | - | Apical meristem |
| sof-miR2098a | osa-miR2098 | 76 | -25.68 | **UCCC**GUGGAGGCAGCCGAUG | 4 | 20 | CA202553 | 5′ | 70 | + | Inflorescence |
| sof-miR2098b | osa-miR2098 | 97 | -47.30 | **UCCC**GUGGAGGCAGCCGAUG | 4 | 20 | CA202553 | 3′ | 70 | + | Inflorescence |
| sof-miR2118a | sbi-miR2118 | 77 | -28.70 | GG**A**AU**A**GGAACAUG**A**AGGAA**A**G | 4 | 22 | CA250529 | 5′ | 41 | + | Inflorescence |
| sof-miR2118b | sbi-miR2118 | 81 | -32.10 | UUCCUGAUGCCUCC**U**AU**U**CCUA | 2 | 22 | CA250529 | 3′ | 46 | + | Inflorescence |
| sof-miR2120a | osa-miR2120 | 124 | -91.40 | ACACCAACCG**A**GACUAAA-**C** | 3 | 19 | CA284264 | 5′ | 47 | + | Seeds |
| sof-miR2120b | osa-miR2120 | 48 | -33.30 | U**C**UAGUC**C**CGGUUGG**A**G**C**UA | 4 | 20 | CA236509 | 3′ | 55 | + | Seeds |
| sof-miR2120c | osa-miR2120 | 43 | -26.50 | **AAA**GAUCUUUAGUCCCGG**U**UUGUUC | 4 | 25 | EC325108 | 5′ | 40 | - | Stem |
| sof-miR2926a | osa-miR2926 | 76 | -28.80 | **AGG**UCGUCGACGUUGGUGCU | 3 | 20 | DV641466 | 5′ | 60 | + | Root |
| sof-miR2926b | osa-miR2926 | 63 | -19.30 | **A**GG**C**CGUCG**UU**GUUGGUGCU | 4 | 20 | CA197829 | 3′ | 60 | + | Seedlings |
| sof-miR5025a | ath-miR5025 | 72 | -32.30 | **ACUG**UAUAUAUGUAAGUGACA | 4 | 21 | CA105537 | 5′ | 29 | + | Seedlings |
| sof-miR5025b | ath-miR5025 | 56 | -23.50 | **ACUG**UAUAUAUGUAAGUGACA | 4 | 21 | CA105537 | 3′ | 29 | + | Seedlings |
| sof-miR5048a | hvu-miR5048a | 51 | -12.20 | UAUUUG**A**AGGUUU**G**AGGUC**A**A**G** | 4 | 22 | CA085151 | 3′ | 36 | - | Apical meristem |
| sof-miR5048b | tae-miR5048 | 53 | -10.90 | UU**A**GCAGGUUUUAGGUC**C**AAGU | 2 | 22 | CA075633 | 3′ | 41 | - | Apical meristem |
| sof-miR5075a | osa-miR5075 | 110 | -41.10 | UUCUCCGUCGC**GU**CCGUC**A**G**G** | 4 | 21 | CA151240 | 5′ | 67 | + | Shoot-root |
| sof-miR5075b | osa-miR5075 | 175 | -72.70 | UUCUCCGUCGC**GU**CCGUCCG**G** | 3 | 21 | CA189415 | 3′ | 71 | + | Lateral buds |
| sof-miR5075c | osa-miR5075 | 110 | -40.10 | UUCUCCGUCGC**GU**CCGUC**A**G**G** | 4 | 21 | CA104318 | 5′ | 67 | - | Seedlings |
| sof-miR5075d | osa-miR5075 | 144 | -70.00 | UUCUCCGUCGC**GU**CCGUC**A**G**G** | 4 | 21 | CA104318 | 3′ | 67 | - | Seedlings |
| sof-miR5168a | ata-miR5168 | 103 | -43.70 | **GGGU**UGUUGUCUGGUUCAAGG | 4 | 21 | CN607727 | 5′ | 52 | + | Leaf |
| sof-miR5168b | ata-miR5168 | 115 | -51.90 | UCGGACCAGGCUUCA**U**UCCC**U** | 2 | 21 | CN607727 | 3′ | 57 | + | Leaf |
| sof-miR5181a | ata-miR5181 | 140 | -81.50 | **C**ACUUAU**AA**UUUGGA**A**CGGAG | 4 | 21 | CA215868 | 3′ | 38 | + | Inflorescence |
| sof-miR5181b | ata-miR5181 | 65 | -48.60 | CACUUAUUU**G**UGGAC-GGAGG | 2 | 20 | CA167807 | 3′ | 50 | - | Stalk Bark |
| sof-miR5281a | bdi-miR5281a | 141 | -78.10 | **A**CUUAUAA**U**U**UA**GAACGGAGG | 4 | 21 | CA141705 | 3′ | 33 | + | Root |
| sof-miR5281b | bdi-miR5281a | 143 | -47.60 | UCUUAUAA**U**U**U**GGAACGGAGG | 2 | 21 | CA101110 | 3′ | 38 | - | Inflorescence |
| sof-miR5281c | bdi-miR5281b | 139 | -60.90 | UCUUAUA**GU**U**U**GGAA**U**GGAGG | 4 | 21 | CF574551 | 3′ | 38 | - | Stem |
| sof-miR5337a | osa-miR5337a | 121 | -60.40 | AAAUU**GUAA**GUCGUUCUAGCU | 4 | 21 | CA296792 | 5′ | 33 | + | Seeds |
| sof-miR5337b | osa-miR5337b | 125 | -43.50 | CUAGAACG**A**C**UUA**CAAUUUGA | 4 | 21 | CA296863 | 3′ | 33 | + | Seeds |
| sof-miR5337c | osa-miR5337a | 121 | -60.40 | AAAUU**GUAA**GUCGUUCUAGCU | 4 | 21 | CA296863 | 5′ | 33 | - | Seeds |
| sof-miR5337d | osa-miR5337b | 125 | -43.50 | CUAGAACG**A**C**UUA**CAAUUUGA | 4 | 21 | CA296792 | 3′ | 33 | - | Seeds |
| sof-miR5502a | osa-miR5502 | 45 | -22.50 | **UAC**GGAUACGGAUACGC**G**GAUAC | 4 | 23 | CA065014 | 3′ | 52 | + | Seedlings |
| sof-miR5502b | osa-miR5502 | 45 | -20.90 | **UAC**GGAUACGGAUACGC**G**GAUAC | 4 | 23 | CA219949 | 3′ | 52 | - | Inflorescence |
| sof-miR5564c | sbi-miR5564c | 78 | -34.40 | ACGCGAGCUGUUUGGCGAAUU | 0 | 21 | CN607542 | 3′ | 52 | + | Leaf |
| sof-miR5568a | sbi-miR5568a | 85 | -42.80 | CA**A**A**AA**GACUUA**U**AAUUUGGA | 4 | 21 | CN608955 | 3′ | 24 | + | Leaf |
| sof-miR5568b | sbi-miR5568a | 84 | -49.50 | CAGAGC**A**ACUUA**U**AAUUUGGA | 2 | 21 | CA119706 | 3′ | 33 | - | Leaf |
| sof-miR5568c | sbi-miR5568c | 133 | -53.20 | UC**CA**UUCCAAAUUGUAAGU**UA** | 4 | 21 | CA147083 | 5′ | 29 | + | Root |
| sof-miR5568d | sbi-miR5568c | 137 | -61.10 | UC**CA**UUCCAAAUUGUAAGU**U**G | 3 | 21 | CA217608 | 5′ | 33 | - | Seedlings |
| sof-miR5831 | osa-miR5831 | 170 | -35.60 | UAGUCAAACUUA**AG**AUAGUU**U**GA**U** | 4 | 24 | CA205395 | 3′ | 25 | - | Stalk Bark |
| sof-miR6164b | nta-miR6164b | 143 | -65.80 | **UCAC**AUAAAUUGAAACGGAGG | 4 | 21 | CA129539 | 3′ | 38 | - | Leaf |
| sof-miR6192 | hvu-miR6192 | 77 | -39.80 | **U**AGGAG**G**G**A**GGGGAAGGGAUCU | 3 | 22 | CA119738 | 3′ | 59 | - | Leaf |
| sof-miR6220a | sbi-miR6220 | 251 | -84.00 | CUCC**G**UCCUAAAUUAUAAGACAUU | 1 | 24 | CA283028 | 5′ | 33 | + | Seeds |
| sof-miR6220b | sbi-miR6220 | 253 | -87.30 | AUGCCUUAUAAUUU**AAA**AUGGAG**G** | 4 | 24 | CA283028 | 3′ | 29 | + | Seeds |
| sof-miR6220c | sbi-miR6220 | 251 | -111.5 | CUCCAU**U**CUAAAUUAUAAG**G**CAUU | 2 | 24 | CA287554 | 5′ | 29 | - | Seeds |
| sof-miR6220d | sbi-miR6220 | 253 | -114.8 | AUGCCUUAUAAUUUGGGAUGGAG**G** | 1 | 24 | CA287554 | 3′ | 42 | - | Seeds |
| sof-miR6225a | sbi-miR6225 | 303 | -131.8 | AA**U**UAGACUCAAAA**UG**UUC**G**UCUC | 4 | 24 | CA120709 | 5′ | 43 | - | Leaf |
| sof-miR6225b | sbi-miR6225 | 303 | -131.8 | GA**G**ACGAAUCUUUU**G**AG**C**CUA**G**UU | 4 | 24 | CA120709 | 3′ | 42 | - | Leaf |
| sof-miR6235 | sbi-miR6235 | 155 | -31.03 | A**U**CG**U**ACAGUAUUUUUCUCU**C**ACA | 3 | 24 | CA188033 | 5′ | 33 | - | Apical meristem |
| sof-miR6249a | osa-miR6249a | 129 | -68.00 | CG**C**G**CC**GAGCUCGCCGGCGGC | 3 | 21 | CA221967 | 5′ | 91 | - | Inflorescence |
| sof-miR6249b | osa-miR6249a | 57 | -22.60 | CG**C**GA**C**G-GCUCGC**G**GGCGGC | 4 | 20 | CA220624 | 3′ | 90 | - | Inflorescence |
| sof-miR6332a | sbi-miR6332a | 102 | -58.40 | GUCGCUUUGACUUUUUUGG**A**ACAU | 1 | 24 | CA131470 | 5′ | 38 | + | Root |
| sof-miR6332b | sbi-miR6332a | 96 | -51.40 | U**A**GAUG**A**ACCAAAA**U**AGUCAAAGC | 3 | 24 | CA131470 | 3′ | 33 | + | Root |
| sof-miR6332c | sbi-miR6332a | 102 | -65.70 | GUCGCUUUGACU**A**UUUUGGU**U**CAU | 2 | 24 | CA131470 | 5′ | 38 | - | Root |
| sof-miR6332d | sbi-miR6332a | 96 | -58.70 | UGGAUGU**U**CCAAAAAAGUCAAAGC | 1 | 24 | CA131470 | 3′ | 38 | - | Root |
| sof-miR7768a | bdi-miR7768a | 91 | -51.94 | CGGCGCCGUCC**C**CGAC**G**GGGAG | 2 | 22 | CA211894 | 5′ | 86 | + | Seedlings |
| sof-miR7768b | bdi-miR7768a | 72 | -30.20 | GCGGCGCCG**G**CCUCGACC**UU**GA | 3 | 22 | CA158925 | 5′ | 77 | - | Shoot-root |
| sof-miR7768c | bdi-miR7768b | 47 | -24.90 | **G**CCGG-CGAGGACGGCCC**U**GC | 3 | 20 | CA198051 | 5′ | 85 | - | Seedlings |
| sof-miR7768d | bdi-miR7768a | 120 | -71.10 | CGGCG**G**CGUCC**A**CG**G**CCGG**C**AG | 4 | 22 | CA201466 | 3′ | 86 | - | Inflorescence |
| sof-miR8005a | stu-miR8005a | 85 | -18.20 | UUUAG**G**GUUUAAGGUUUAG**G**GUUU | 2 | 24 | CA297572 | 5′ | 33 | - | Seeds |
| sof-miR8005b | stu-miR8005a | 91 | -15.10 | UUUAG**G**GUUUAAGGUUUAG**G**GUUU | 2 | 24 | CA297572 | 3′ | 33 | - | Seeds |
| sof-miR8005c | stu-miR8005a | 141 | -34.20 | UUUAG**G**GUUUAAGGUUUAG**G**GUUU | 2 | 24 | CA297572 | 5′ | 33 | - | Seeds |
| sof-miR8005d | stu-miR8005a | 147 | -32.20 | UUUAG**G**GUUUAAGGUUUAG**G**GUUU | 2 | 24 | CA297572 | 3′ | 33 | - | Seeds |
| sof-miR9780 | tae-miR9780 | 95 | -54.00 | **G**G**C**GUCGGCGCUGCACGCGGC | 2 | 21 | CA150941 | 5′ | 86 | + | Shoot-root |
